# Trends in Shoulder Arthroplasty in Germany: A 10-Year Epidemiological Analysis of Patients with Primary Osteoarthritis of the Shoulder

**DOI:** 10.3390/healthcare12090949

**Published:** 2024-05-06

**Authors:** Felix Frederik Hochberger, Philipp Herrmann, Maximilian Rudert, Kilian List, Ioannis Stratos

**Affiliations:** Department of Orthopaedic Surgery, Julius-Maximilians University Wuerzburg, Koenig-Ludwig-Haus, Brettreichstrasse 11, 97074 Wuerzburg, Germany; felix.hochberger@klh.de (F.F.H.); philipp.herrmann@klh.de (P.H.); m-rudert.klh@uni-wuerzburg.de (M.R.); k-list.klh@uni-wuerzburg.de (K.L.)

**Keywords:** reverse total shoulder arthroplasty, cuff-tear arthropathy, age distribution, healthcare data

## Abstract

Shoulder arthroplasty has significantly gained popularity in orthopedic surgery, driven by progress in prosthesis design and surgical techniques. This study explored the epidemiology of shoulder arthroplasty, analyzing healthcare data from 2012 to 2022 for primary osteoarthritis of the shoulder. The data included patient demographics and types of surgical procedures. Data analysis indicates a higher utilization rate of reverse total shoulder arthroplasty (RTSA; *n* = 41,251) over total- (TSA; *n* = 18,679) and hemiarthroplasty (HSA; *n* = 12,827) for primary shoulder osteoarthritis. Overall, a significant increase in RTSA procedures from *n* = 2237 (2012) to *n* = 5415 (2022) was observed, representing more than a two-fold increase of 121.1%. The relative proportion of RTSA among all types of shoulder arthroplasty increased from 39% (2012) to 68.6% (2022), while HSA decreased and TSA essentially remained constant. Age analysis identified the following peaks: RTSA, 77 ± 7 y; HSA, 68 ± 12 y; and TSA, 67 ± 10 y. Among the over 60s, significantly more women were treated with any type of prosthesis, whereas in young patients (45 to 59 y), more men received HSA or TSA. Our study confirms that RTSA has become the preferred choice for elderly patients in Germany, reflecting the prevailing preference despite varying patient ages and conditions, with a noted difference in sex in treatment prevalence.

## 1. Introduction

Osteoarthritis (OA) of the shoulder is a cause of pain, limited range of motion, and impaired function among the elderly [1]. Although shoulder OA is not as common as OA of the hip or knee, it has been shown in cadaver and radiographic studies to affect up to 32.8% of people over the age of 60 [2,3]. As life expectancy increases and the demands of our aging society for unrestricted quality of life into advanced age rise, implantation rates have been rising worldwide [4,5]. The 2020 annual report of the DVSE (German Shoulder and Elbow Society) arthroplasty register shows an overall increase in the number of operations from 2006 to 2019 with relatively higher growth in reverse shoulder arthroplasty (RTSA) among all shoulder prostheses and a decrease in hemiarthroplasty (HSA) (RTSA: 2006: *n* = 72—2019: *n* = 1018; TSA: 2006: *n* = 70—2019: *n* = 250; HSA: 2006: *n* = 76—2019: *n* = 37) [6]. Several studies have explored the epidemiologic trends of shoulder arthroplasty on a national and international level [7,8,9,10,11,12,13,14]. A recently published epidemiological review from 2022 demonstrated a substantial increase in operation numbers of shoulder arthroplasty in recent years at a greater rate than total knee and hip arthroplasty, largely because of the exponential increases in RTSA, with projections continuing to rise over the next decade [15]. However, there is a noticeable gap in research examining the specific details of primary osteoarthritis arthroplasty procedures over a longer period, particularly concerning the types of shoulder arthroplasties performed and their correlation with patient age. Although anatomic total shoulder arthroplasty (TSA) is conventionally preferred for patients with intact rotator cuff [16,17], reverse total shoulder arthroplasty (RTSA) has been demonstrated to be a viable option for patients with glenoid bone loss and intact rotator cuff function who may be elderly and less active [18]. Consequently, both anatomical and demographic factors appear to have a substantial impact on the choice of prosthesis. Moreover, it remains largely uncertain in which patient group the hemiprosthesis (HSA) would be considered preferable over TSA. Several authors reported on the lack of high-quality studies focusing on patients treated with shoulder arthroplasty with regard to demographic analyses [19,20]. The authors of a recently published Cochrane review by Craig et al. analyzed the existing literature for studies comparing the different types of shoulder arthroplasty. Despite not looking in detail at demographic aspects, they concluded that, to date, it still remains uncertain which type of shoulder prosthesis is best in different situations [21]. This problem appears to be most evident in the group of patients over 75 years of age, in which anatomical and reverse total shoulder arthroplasty appear to be overlapping in use regardless of the underlying indication. In order to take a closer look at this “gray area”, this study aims to examine the use of shoulder arthroplasty in Germany in the different age groups from a demographic point of view for patients with OA. A sex preference for shoulder arthroplasty is also described in current literature. A recent study from Italy showed that shoulder arthroplasty is significantly more common among female patients than males [10]. Similarly, a Finnish study spanning from 2004 to 2015 showed that women underwent significantly more primary shoulder arthroplasties than men [9]. It is unclear if this trend is also present in Germany.

This study aimed to investigate the current state of shoulder arthroplasty for OA in Germany with respect to the use of RTSA, TSA, or HSA within the context of epidemiological data (sex and age) and their development over time. In particular, patient age at the time of implantation may be of considerable interest, as the decision on the correct choice of prosthesis may rather be made by the considerable durability of implants than on patients’ radiological findings.

## 2. Materials and Methods

### 2.1. Data Source and Data Structure

For this study, patient hospital billing data were sourced from the Federal Statistical Office of Germany, covering the period from 2012 to 2022. Our inclusion criteria focused on patients diagnosed with primary arthrosis of the shoulder, indicated by the ICD-10 diagnosis code M19.0*. Additionally, these patients must have undergone a surgical procedure related to shoulder arthroplasty, as specified by the OPS codes (Operations and Procedures Key in the German healthcare system) 5-824.0*, 5-824.1, and 5-824.2*, within the same hospital admission. An authorized representative from the Federal Statistical Office of Germany provided the data, which was specifically requested and purchased for our research needs. The data set encompasses various parameters, including the diagnosis year (spanning 2012–2022), sex (categorized as male and female), the relevant OPS codes, and age of the patients, categorized into five-year age groups. The format of the data is aggregated and offers open access at the following URL: https://github.com/ioannis-stratos/shoulder (accessed on 20 April 2024). 

### 2.2. Data Processing

In this study, data conversion from a wide to a long format was conducted utilizing R software (version 2023.12.0, R-Studio, Boston, MA, USA) along with the ‘reshape2’ package. For the purpose of subgroup analysis, OPS codes 5-824.0, 5-824.0x, 5-824.1, 5-824.00, and 5-824.01 were utilized for HSA; 5-824.2 and 5-824.20 for categorizing TSA; and 5-824.21 for RTSA. Additional subgroup categorization was based on sex, year of diagnosis, and patient age, which was segmented into four age groups: “up to 44”, “45–59”, “60–79”, and “over 80” years. The computation and analysis of these subgroups were executed using Tableau Desktop (version 2023.3, Tableau Software, Seattle, WA, USA), and the results were organized into tables for further examination.

### 2.3. Statistical Analysis

Linear regression analyses were conducted using GraphPad Prism software (version 10.1.1; GraphPad Software, San Diego, CA, USA), through which corresponding graphical representations were also generated. The overall significance of the linear regression models was evaluated using the F-test to determine if the models significantly deviated from a null hypothesis of zero effect. For all regression analyses, the independent variable was consistently time, spanning from 2012 to 2022, and is represented on the x-axis. The dependent variable was always depicted on the y-axis. For calculation, Gaussian distributions were utilized for the purposes of nonlinear regression analysis. Group distribution differences were statistically examined employing the chi-squared test. The “Joinpoint Trend Analysis Software” version 5.1.0 from the National Institutes of Health (Bethesda, MD, USA) was employed to perform the join point regression analysis. A threshold of *p* ≤ 0.05 was defined for the determination of statistical significance in all analytical procedures.

## 3. Results

### 3.1. Overall Results

**Figure 1:**
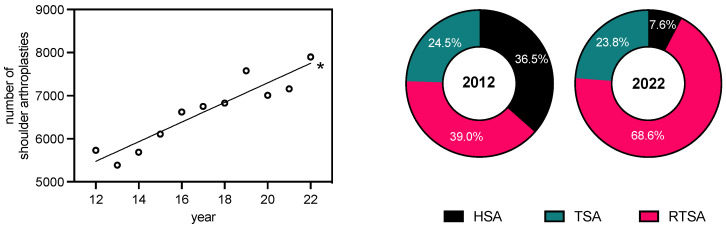
Linear regression analysis of primary osteoarthritis cases post-endoprosthesis per year for the period 2012–2022 (R^2^: 0.877; y = 227.6x + 2746; F: 64.11; *: slope of the line significantly different from zero, evidenced by an F-statistic, *p* < 0.0001). The data are categorized based on the type of endoprosthesis used (HSA, TSA, and RTSA) for the years 2012 and 2022.

**Figure 2:**
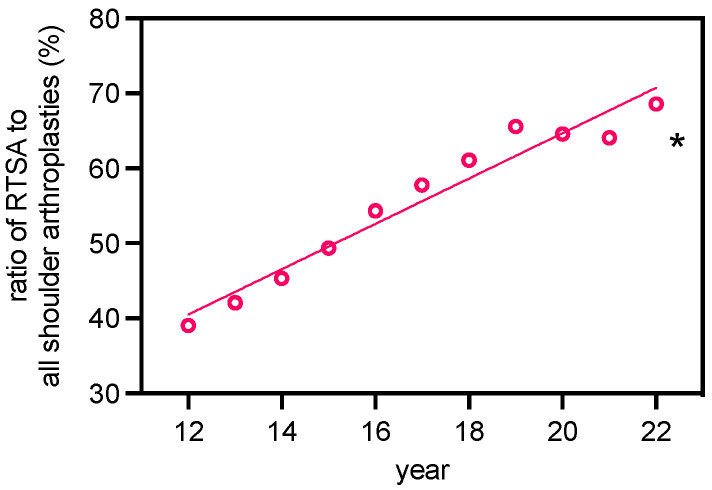
Linear regression analysis of the ratio of RTSA to all shoulder arthroplasties of primary osteoarthritis cases for the period 2012–2022 (R^2^: 0.9502; y =3.02x + 4.19; F: 171.80; *: slope of the line significantly different from zero, evidenced by an F-statistic, *p* < 0.0001).

In total, we identified 72,757 arthroplasty procedures (TSA, RTSA, or HSA) performed in Germany between 2012 and 2022 for patients diagnosed with primary osteoarthritis of the shoulder (OA). Substantially more RTSAs (*n* = 41,251) than TSAs (*n* = 18,679) and HSAs (*n* = 12,827) were carried out over the entire period. A significant increase in RTSA was observed over the 10 years examined (2012: *n* = 2237; 2022: *n* = 5415; y = 322.7x − 1736; R^2^ = 0.9397; F = 140.2; slope with *p* < 0.0001 non-zero), which corresponds to an increase of 260%. In terms of TSA (2012: *n* = 1402; 2022: *n* = 1881; y = 46.7x + 905; R^2^ = 0.7691; F = 30.0; slope with *p* < 0.001 non-zero), we could not identify any major change, while HSA decreased in this context (2012: *n* = 2091; 2022: *n* = 603; y = 322.7x − 1736; R^2^ = 0.9445; F = 153.3; slope with *p* < 0.0001 non-zero). This represents a decline of 71%. Over the entire period, the number of HSAs consistently remained below that of TSA. Overall, the relative ratio of RTSA to all types of shoulder arthroplasty increased from 39% (2012) to 68.6% (2022), whereas HSAs were implanted significantly less in relative terms (2012: 36.5%; 2022: 7.6%). However, the proportion of TSA among all types of shoulder arthroplasty remained largely constant over the observation period (2012: 24.5%; 2022: 23.8%). The relative incidence distribution of RTSA, TSA, and HSA among all types of shoulder arthroplasty over the period studied is illustrated in Figure 1. The increase in RTSA over the years followed a linear growth pattern (Figure 2). Analysis of join point regression revealed that the total number of shoulder arthroplasties increased significantly, particularly from 2012 to 2018 (inflection point in 2018). A similar trend was observed for the RTSA and the percentage of RTSAs from 2012 to 2019 (inflection point in 2019). For TSA, a significant increase was noted until 2016 (inflection point in 2016), while the number of HSAs significantly decreased between 2012 and 2018 (inflection point in 2018). For more details, see Suppl. Figure S1.

### 3.2. Age Distribution

**Figure 3:**
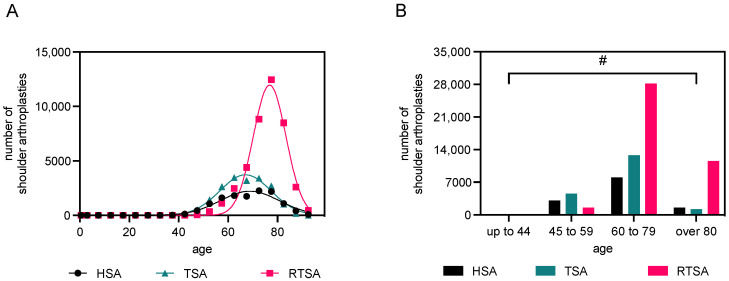
Figure (**A**) presents a Gaussian regression analysis of all cases of primary osteoarthritis following shoulder arthroplasty from 2012 to 2022. The data are categorized based on the type of arthroplasty used and the patient’s age. The calculated mean age and standard deviation (SD) were for the hemiprosthesis, 68.8 ± 12.0 years; for the total shoulder arthroplasty, 67.0 ± 10.3 years; and for the reverse shoulder arthroplasty, 76.8 ± 12.0 years. Figure (**B**) presents categorized data for all cases of primary osteoarthrosis following endoprosthesis implantation from 2012 to 2022. The data are grouped based on the type of endoprosthesis used and the patient’s age. Data for the “up to 44 years” age group: 144 HSA; 142 TSA; and 9 RTSA (values too low to be visualized effectively). # Chi-squared test; *p* < 0.0001, indicating a statistically significant association between patient age groups and the types of endoprosthesis used.

**Figure 4:**
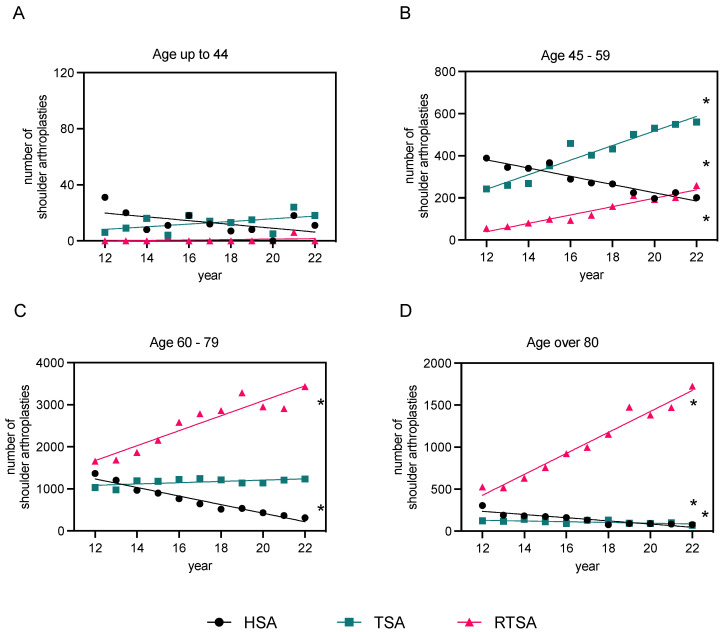
Linear regression analysis of primary osteoarthritis cases post-endoprosthesis in males and females, 2012–2022. The data are categorized based on the type of endoprosthesis used (HSA, TSA, and RTSA) and patient age groups ((**A**): “<44” years, (**B**): “45–59” years, (**C**): “60–79” years, and (**D**): “>80” years of age). Refer to Supp. Table S1 for detailed regression analysis parameters. The level of significance is shown in Supp. Table S1 (* *p*-value < 0.05), indicating if each slope is significantly non-zero.

Overall, the mean age for HSA was 68.8 ± 12.0 years, 67.0 ± 10.3 years for TSA, and 76.8 ± 12.0 years for RTSA, demonstrating a predominantly higher age group among RTSA patients (Figure 3A). Considering the distribution patterns of implantation rates for different shoulder arthroplasties (RTSA, HSA, and TSA) across the age groups (<44, 45–59, 60–79, and >80), significant disparities in distribution are evident (Figure 3B). The analysis revealed that the implantation rates for RTSA, HSA, and TSA differ significantly among the age groups, as confirmed by the χ^2^ test. In the 75–80 years age group, RTSAs were by far the most commonly used (*n* = 12,459) and are consistently outnumbered by other implant types from a patient age of >65 years. Among patients younger than 44 years of age, the following number of implantations was identified: HSA: *n* = 144; TSA: *n* = 142; RTSA: *n* = 9. HSAs peak between 60 and 75 years; however, RTSAs are still superior in this age range. Age distribution in relation to type of arthroplasty is demonstrated in Figure 4A–D.

### 3.3. Distribution of Sex

**Figure 5:**
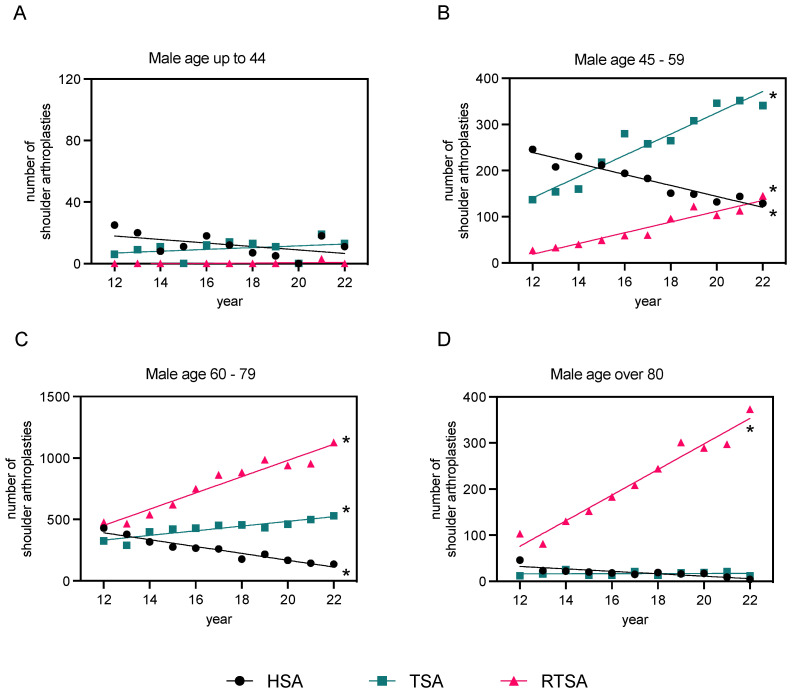
Linear regression analysis of primary osteoarthritis cases post-endoprosthesis in males, 2012–2022. The data are categorized based on the type of endoprosthesis used (HSA, TSA, and RTSA) and patient age groups ((**A**): “<44” years, (**B**): “45–59” years, (**C**): “60–79” years, and (**D**): “>80” years of age). Refer to Supp. Table S1 for detailed regression analysis parameters. The level of significance is shown in Supp. Table S2 (* *p*-value < 0.05), indicating if each slope is significantly non-zero.

**Figure 6:**
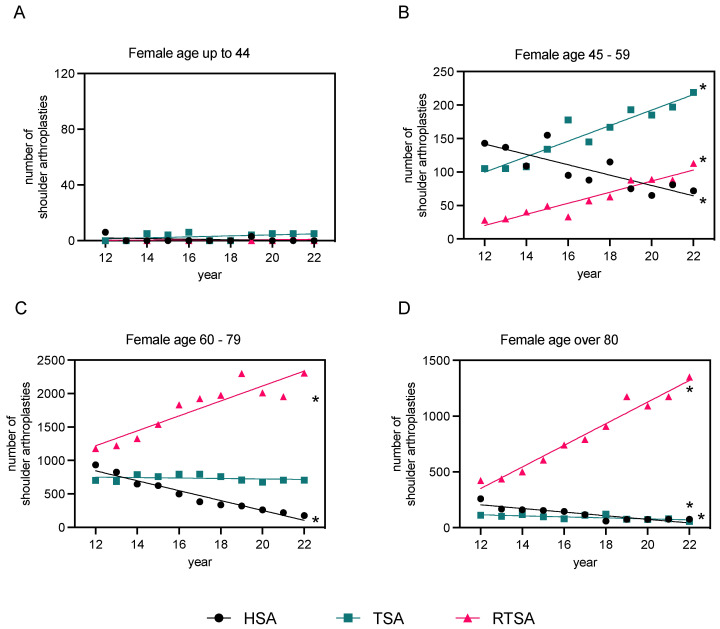
Linear regression analysis of primary osteoarthritis cases post-endoprosthesis in females, 2012–2022. The data are categorized based on the type of endoprosthesis used (HSA, TSA, and RTSA) and patient age groups ((**A**): “<44” years, (**B**): “45–59” years, (**C**): “60–79” years, and (**D**): “>80” years of age). Refer to Supp. Table S3 for detailed regression analysis parameters. The level of significance is shown in Supp. Table S3 (* *p*-value < 0.05), indicating if each slope is significantly non-zero.

Looking at the data across all age groups, the overall rate of shoulder arthroplasty (HSA, TSA, and RTSA) was 1.9 times higher in women than in men (48,036 women to 24,721 men). Below the age of 60, the age-standardized ratio was higher for men in HSA and TSA but not in RTSA. Below the age of 60, the age-standardized ratio was higher for men in HSA and TSA, while among the over 60s, the age-standardized ratio was higher for women in all three types of arthroplasties. Within the young patients (“<44” years), only men were treated. In the age group “60–79” years in particular, the number of women treated was 1.9 times higher than that of men in HSA, 2.3 times higher in RTSA, and 1.7 times higher in TSA, and therefore broadly balanced in this age range for all three implants. The most significant sex-specific differences were observed in the age group “≥80” years, with up to 6.5 times higher rates for women treated with HSA (Suppl. Table S1). Figure 5 and Figure 6 demonstrate the distribution of sex in relation to the age groups described for all three types of prostheses.

## 4. Discussion

The overall findings of this study reveal a significant rise in shoulder arthroplasty procedures in Germany over the past decade, particularly for RTSA. Accounting for more than half of all cases, RTSA emerged as the most performed procedure. By the end of the period investigated, the instances of RTSA saw a multi-fold rise compared to the starting year. The share of RTSA among all types of shoulder arthroplasty has nearly doubled throughout the observation period, with a consistent yearly increase being noted. Given that this trend is expected to continue, it is likely that numbers will keep heading up in the future. However, the evolution of TSA was less sharp. Despite an observed rise in the number of operations, the difference between 2012 and 2022 was insignificant. In contrast, we saw a clear drop in the number of HSA. Recent studies have shown that RTSA and TSA provide better results than HSA in severe shoulder OA [22,23]. In their retrospective study evaluating TSA and HSA, the research group led by Sandow et al. presented long-term results over 10 years. They demonstrated the superiority of TSA over HSA in terms of pain and function after 2 years in patients with an intact rotator cuff and no deterioration in function or excessive failure rate of the glenoid component after 10 years. Revision from HSA to TSA may be complicated by glenoid erosion. It is likely that the delay in the implantation of HSA in younger patients was due to the assumption that postponing glenoid implantation in shoulder arthroplasty would be a more predictable option and that preservation of the glenoid would be necessary for a possible later revision to TSA. However, the available data on durability and early complications following HSA compared to TSA seem to contradict this assumption [23,24,25]. This finding may justify the increase in TSA and RTSA rates and the decrease in HSA. However, little data are available on the clinical performance of the different types of endoprosthesis so far [11]. In terms of age range, RTSA was by far the most commonly used in patients over 60 years of age. Among these, the ratio of women to men was around 2.4 times higher in the “60–79” age group and even 3.9 times higher in the “over 80” age group. However, a quantitative predominance of TSA over RTSA could only be observed in the groups of “45–59” years and “<44” years. Overall, a distinct shift in the distribution of sex was observed. Irrespective of the type of implant, the rate of male patients was higher among patients below 60 years of age, whereas women were treated far more frequently than men in the over 60s. 

Several studies have already been conducted reporting epidemiological data on the development of shoulder arthroplasty in various countries [7,8,12,13,14]. In fact, most of them reported a sharp increase in shoulder arthroplasty over the past 20 years and even suggested further potential growth in the future. In their epidemiological study, the research group led by Day et al. investigated national trends and forecasts concerning the scope of procedures and prevalence rates for shoulder and elbow arthroplasty in the United States between 2007 and 2013. They analyzed registry data and demonstrated that the volume of arthroplasty procedures of the upper extremity increased annually by 6–13%. Compared to 2007, a further increase in the number of operations from 192% to 322% was predicted up to 2015. Although the authors of the present study focused solely on the trends of various types of shoulder arthroplasty, and Day et al. also considered elbow prostheses, similar findings could be observed in particular for reverse RTSA, with an overall increase in operation numbers of 260% during the period studied from 2012 to 2022 [7]. Similarly, a research group led by Dillon et al. showed an increase in the incidence of shoulder arthroplasty from 6.1% in 2005 to 13.4 in 2013 and a general increase in TSA with a concomitant decrease in HSA for OA. These results differ, at least in part, from the results presented here, given that in our cohort, the number of TSA for OA remained largely constant while HSA also decreased [8]. Furthermore, Schairer et al., in 2015, demonstrated that in the United States, by the year 2011, the relative proportion of RTSA among all shoulder arthroplasties was increasing due to the expansion of indications [13]. Our results support this thesis, with an increasing overall relative ratio of RTSA to all types of shoulder arthroplasty from 39% (2012) to 68.6% (2022). In a similar review to the one conducted by the authors, Westermann et al. found an increase in the use of both RTSA and TSA between 2002 and 2011, while HSA declined. RTSA patients were overall older and more often female [14]. These results are largely consistent with those presented here; however, the increase in TSA was comparatively more modest in our study. A recently published study from Italy demonstrated a three-fold increase in shoulder arthroplasty in a cross-sectional study over the period 2009–2019 and suggested a 72.3% increase in the following ten years [10]. Likewise, national register data confirm the increasing disenrollment. The New Zealand, Australian, Norwegian, and Danish Joint Registries recently identified a relevant rise in implantation rates for shoulder arthroplasty [5,10,26,27]. Our results are largely consistent with the trend in registered cases of the German arthroplasty register of the DVSE, demonstrating an overall increase in shoulder arthroplasty in recent years, in particular among RTSA (2006: *n* = 72—2019: *n* = 1018) and TSA (2006: *n* = 70—2019: *n* = 250) [6]. In this study, the period examined largely overlaps with the report from the DVSE register. However, the German register data used in this study based on OPS coding showed substantially higher operation numbers than those recorded in the DVSE register. In this study, the period examined largely overlaps with the report from the DVSE register. However, the German register data used in this study based on OPS coding showed substantially higher operation numbers than those recorded in the DVSE register. This is most likely due to the fact that the volume of procedures performed in the participating clinics varies widely, and the majority of registered procedures are carried out in just 10 clinics across all of Germany. Therefore, it may be assumed that operation numbers in the DVSE register recorded so far are strongly underpowered. 

In terms of the distribution of sex, the findings of our study are largely consistent with the literature. In their epidemiological study from 2021, Farley et al. examined the prevalence of TSA, RTSA, and HSA in primary OA in the USA with respect to age and distribution of sex [28]. They found that women had a higher prevalence of RTSA and TSA in all age groups compared to male patients. These results have been confirmed by a German research group led by Oppermann et al. [12]. In this study, there was evidence that the ratio of women is higher above 60 years of age, regardless of the implant model. Below the age of 60, men were affected more frequently. Similar findings were made by Westermann et al., showing predominantly older and female patients undergoing RTSA [14]. Looking at the results of the study presented here, it is evident that there was an overall 1.9-fold higher rate of female patients treated with shoulder arthroplasty compared to men over the time period studied. This trend was most noticeable among the patients over 60 years old. In this age group, women were treated more frequently than men with all types of prostheses (RTSA, TSA, and HSA). The most significant sex-specific differences were observed in the age group “≥80” years, with up to 6.5 times higher rates for women treated with HSA. In contrast, patients under the age of 60 who underwent HSA or TSA were predominantly male. In particular, those under the age of 44 who underwent these procedures were consistently male. Overall, the results regarding sex distribution are consistent with previous studies on this topic. However, an explanation for this age-related ratio between men and women who undergo shoulder arthroplasty is yet to be found and requires further investigation. 

Despite the fact that shoulder arthroplasty as the treatment of choice in cases of end-stage primary shoulder OA has long been established, it remains controversial which type of endoprosthesis would be favorable. In particular, the question of whether RTSA or TSA would be the right choice for elderly patients remains unresolved and often depends on the surgeon’s conviction. Both TSA and RTSA are viable treatment options for primary OA. However, TSA has been found to provide improved functional outcomes [29,30] and a good long-term survival rate [29,31]. Other studies have contradicted this statement, describing both RTSA and TSA as equivalent treatment strategies for severe primary OA in elderly patients [32,33]. In a recently published study by Kim et al., the authors demonstrated that both RTSA and TSA achieve good results in severe primary OA; however, TSA provides better clinical outcomes and range of motion in the short to mid-term follow-up period [34]. Nevertheless, there is an increased risk of secondary rotator cuff insufficiency following primary TSA in elderly patients, which is reported in the literature with a prevalence of 1.3% to 16.8% [35,36] and is associated with reduced survival rates and higher rates of revision surgery [37]. However, according to the literature, the incidence of secondary rotator cuff insufficiency was found to increase exponentially with age but not necessarily result in symptoms and failure [38], which might explain the broadly comparable functional outcomes and survival rates of RTSA and TSA. 

The ratio of TSA to RTSA varies by country. In New Zealand, the ratio was 65–35%, in Australia 56–44%, in Denmark 27–73%, and in Norway 23–77% [5,26,27]. In Germany, the ratio appears to be comparable to that in New Zealand, with an average rate of 65–35% observed between 2008 and 2012, which may be largely due to the expanded indications for RTSA [39,40]. RTSA was initially intended for the treatment of rotator cuff pathologies. Under the main coded diagnosis of cuff tear, RTSA still remains the most commonly used shoulder arthroplasty. As surgeons gain more experience with RTSA, the indications are expanding, accounting for an increase of 260% since 2012.

Despite a persistent divergence of overlapping indications, in recent years, substantial progress has also been achieved in TSA as a result of continuous improvements in implant technologies. Advanced stemless and modular implant systems with improved metaphyseal anchoring facilities have led to improved preservation of the metaphyseal bone stock and facilitated conversion to RTSA in revision cases. Available 1-year results indicate promising clinical and functional outcomes [41]. Consequently, there has been a progressive expansion of indications and a reduction in the average age of patients. The trend towards metaphyseal cementless stem fixation has also facilitated potential revision surgeries. Moreover, recently developed metal-back glenoid components with titanium inlays and humeral-side polyethylene implants seem to be able to overcome premature glenoid loosening due to asymmetrical wear [42]. These advancements have led to extended durability, reduced complication rates, and overall improved functional outcomes [43,44]. 

This study is not without limitations. It only provided a demographical overview of arthroplasty procedures performed in Germany and did not address functional outcomes, perioperative complications, prosthesis durability, and differences in surgical technique. Nevertheless, the epidemiological data provided information about demographic changes and the efficacy of specific types of arthroplasties. Furthermore, the data extracted procedures for the ICD code M19.x. It cannot be ruled out that, in some cases, surgeons documented a primary OA (M19.x) instead of a code for secondary OA such as cuff-tear arthropathy, posttraumatic OA, or rheumatoid OA. In addition, the authors deliberately only included diagnoses of primary osteoarthritis of the shoulder but not secondary and cuff-tear arthropathy. This certainly may lead to a considerably higher number of overall RTSA implants in Germany than demonstrated here. However, this was not the primary aim of this study. In addition, we were only able to analyze the main diagnoses and no secondary diagnoses. This means that, depending on the individual coding of diagnoses performed by surgeons, the data presented here may be misinterpreted. Furthermore, the results presented are based on a national report; differences in the choice of type of implant between different centers may affect the outcomes as well. Despite this limitation, this study presents high-quality long-term data illustrating the current development of shoulder arthroplasty in Germany from 2012 to 2022. Due to standardized international OPS codes, an international comparison with other countries based on these data is facilitated.

## 5. Conclusions

Overall, taking into account the period studied, the amount of shoulder arthroplasty in Germany has significantly increased over the past 10 years. HSA seems to be applied increasingly less in patients over 60 years of age. In contrast, TSA and RTSA are still the most frequently performed procedures, and the number of operations continues to rise, with RTSA outnumbering the other types of arthroplasties in patients over 60 years. The incidence of RTSA is expected to increase further in the following years, with its survival rate and durability offering great prospects for the self-sufficiency of the aging population. However, despite the higher utilization rate of RTSA among all types of shoulder arthroplasty over recent years, it is important in this context not to assume that both TSA and HSA no longer have any clinical value in the treatment of shoulder OA. Indeed, the rising number of operations rather confirms the success of ongoing research and development efforts, resulting in continuously improved implants and subsequent advances in patient care. Physicians should, therefore, continue to focus on establishing the correct indication and the correct choice of prosthesis in this context. The implantation rate for women aged 60 years and older is three times higher than for men, especially for TSA and RTSA. The true number of shoulder arthroplasties performed in Germany each year outreaches the cases that are actually evaluated in the German Shoulder Registry of DVSE by far. This illustrates the great scientific treasure buried and once more underlines the importance of a nationwide shoulder arthroplasty registry. The findings of this study support further investigations comparing both the clinical and functional effectiveness of the different types of prosthesis and analyzing health economic differences as well as the respective burden on the healthcare system depending on the type of prosthesis.

## Data Availability

Data are available in a publicly accessible repository.

## Supplementary Materials

The following supporting information can be downloaded at: https://www.mdpi.com/article/10.3390/healthcare12090949/s1, Table S1: Linear regression analysis parameters for primary osteoarthritis cases in males and females post-endoprosthesis, 2012–2022; Table S2: Linear regression analysis parameters for primary osteoarthritis cases in males post-endoprosthesis, 2012–2022; Table S3: Linear regression analysis parameters for primary osteoarthritis cases in females post-endoprosthesis, 2012–2022; Figure S1: Joint Point Regression Analysis.
