# Supplementary material for: Trends in Shoulder Arthroplasty in Germany: A 10-Year Epidemiological Analysis of Patients with Primary Osteoarthritis of the Shoulder
Source: Healthcare (Basel). 2024 May 6;12(9):949. doi: 10.3390/healthcare12090949 (PMC11083230; doi:10.3390/healthcare12090949)
Supplement: Supplementary file 1 [file healthcare-12-00949-s001.zip › healthcare-2930099-supplementary.pdf]

**Supplementary Material**

| Male and Female | HSA | TSA | RTSA |
|---|---|---|---|
| Age up to 44 | | | |
| $R^2$ | 0.2938 | 0.249 | 0.07826 |
| F | 3.745 | 2.984 | 0.7642 |
| P value | 0.085 | 0.1182 | 0.4048 |
| Equation | y=-1.355x+36.12 | y=0.9455x-3.164 | y=0.1636x-1.964 |
| Age 45-59 | | | |
| $R^2$ | 0.9043 | 0.9302 | 0.9323 |
| F | 85 | 119.8 | 123.8 |
| P value | <0.0001 | <0.0001 | <0.0001 |
| Equation | y=-19.62x+616.6 | y=34.65x-174.9 | y=19.95x-200.4 |
| Age 60-79 | | | |
| $R^2$ | 0.9486 | 0.3524 | 0.8858 |
| F | 166.1 | 4.897 | 69.82 |
| P value | <0.0001 | 0.0542 | <0.0001 |
| Equation | y=-102.0x+2461 | y=15.48x+898.4 | y=177.9x-464.0 |
| Age over 80 | | | |
| $R^2$ | 0.8053 | 0.4324 | 0.9663 |
| F | 37.22 | 6.855 | 258.2 |
| P value | 0.0002 | 0.0279 | <0.0001 |
| Equation | y=-18.87x+463.5 | y=-4.427x+184.8 | y=124.7x-1070 |

Table S1: Linear regression analysis parameters for primary osteoarthritis cases in males and females post-endoprosthesis, 2012-2022.

This is a supplementary table to Figure 4. $R^2$ represents the coefficient of determination, and F denotes the F-statistic. The P value indicates the level of significance, showing whether the slope of the regression line is significantly different from zero. The slope coefficient in the equation indicates the calculated increase or decrease in cases per year.

| Male | HSA | TSA | RTSA |
|---|---|---|---|
| Age up to 44 | | | |
| $R^2$<br>F<br>P value<br>Equation | 0.263<br>3.211<br>0.1067<br>y=-1.136x+31.59 | 0.1138<br>1.155<br>0.3104<br>y=0.5909x-0.2273 | 0.16<br>1.714<br>0.2229<br>y=0.1091x-1.582 |
| Age 45-59 | | | |
| $R^2$<br>F<br>P value<br>Equation | 0.915<br>96.83<br><0.0001<br>y=-11.88x+381.9 | 0.9254<br>111.6<br><0.0001<br>y=23.05x-135.5 | 0.9232<br>108.2<br><0.0001<br>y=11.65x-121.1 |
| Age 60-79 | | | |
| $R^2$<br>F<br>P value<br>Equation | 0.9284<br>116.7<br><0.0001<br>y=-27.86x+725.9 | 0.8234<br>41.98<br>0.0001<br>y=19.00x+104.3 | 0.9397<br>140.2<br><0.0001<br>y=66.10x-341.6 |
| Age over 80 | | | |
| $R^2$<br>F<br>P value<br>Equation | 0.6793<br>19.06<br>0.0018<br>y=-2.618x+63.51 | 0.006593<br>0.05973<br>0.8124<br>y=0.1091x+14.78 | 0.9592<br>211.5<br><0.0001<br>y=27.73x-256.7 |

**Table S2: Linear regression analysis parameters for primary osteoarthritis cases in males post-endoprosthesis, 2012-2022.**

This is a supplementary table to Figure 5. $R^2$ represents the coefficient of determination, and F denotes the F-statistic. The P value indicates the level of significance, showing whether the slope of the regression line is significantly different from zero. The slope coefficient in the equation indicates the calculated increase or decrease in cases per year.

| Female | HSA | TSA | RTSA |
|---|---|---|---|
| Age up to 44 | | | |
| $R^2$ | 0.1391 | 0.2198 | 0.02222 |
| F | 1.455 | 2.535 | 0.2045 |
| P value | 0.2585 | 0.1458 | 0.6618 |
| Equation | y=-0.2182x+4.527 | y=0.3545x-2.936 | y=0.05455x-0.3818 |
| Age 45-59 | | | |
| $R^2$ | 0.6857 | 0.8949 | 0.9022 |
| F | 19.63 | 76.65 | 83.07 |
| P value | 0.0016 | <0.0001 | <0.0001 |
| Equation | y=-7.736x+234.7 | y=11.60x-39.38 | y=8.291x-79.31 |
| Age 60-79 | | | |
| $R^2$ | 0.9443 | 0.0673 | 0.8431 |
| F | 152.6 | 0.6494 | 48.36 |
| P value | <0.0001 | 0.4411 | <0.0001 |
| Equation | y=-74.09x+1735 | y=-3.518x+794.1 | y=111.8x-122.4 |
| Age over 80 | | | |
| $R^2$ | 0.7922 | 0.5074 | 0.9656 |
| F | 34.3 | 9.272 | 252.5 |
| P value | 0.0002 | 0.0139 | <0.0001 |
| Equation | y=-16.25x+400.0 | y=-4.536x+170.0 | y=96.97x-812.8 |

Supplementary Table S3: Linear regression analysis parameters for primary osteoarthritis cases in females post-endoprosthesis, 2012-2022.

This is a supplementary table to Figure 6. $R^2$ represents the coefficient of determination, and F denotes the F-statistic. The P value indicates the level of significance, showing whether the slope of the regression line is significantly different from zero. The slope coefficient in the equation indicates the calculated increase or decrease in cases per year.

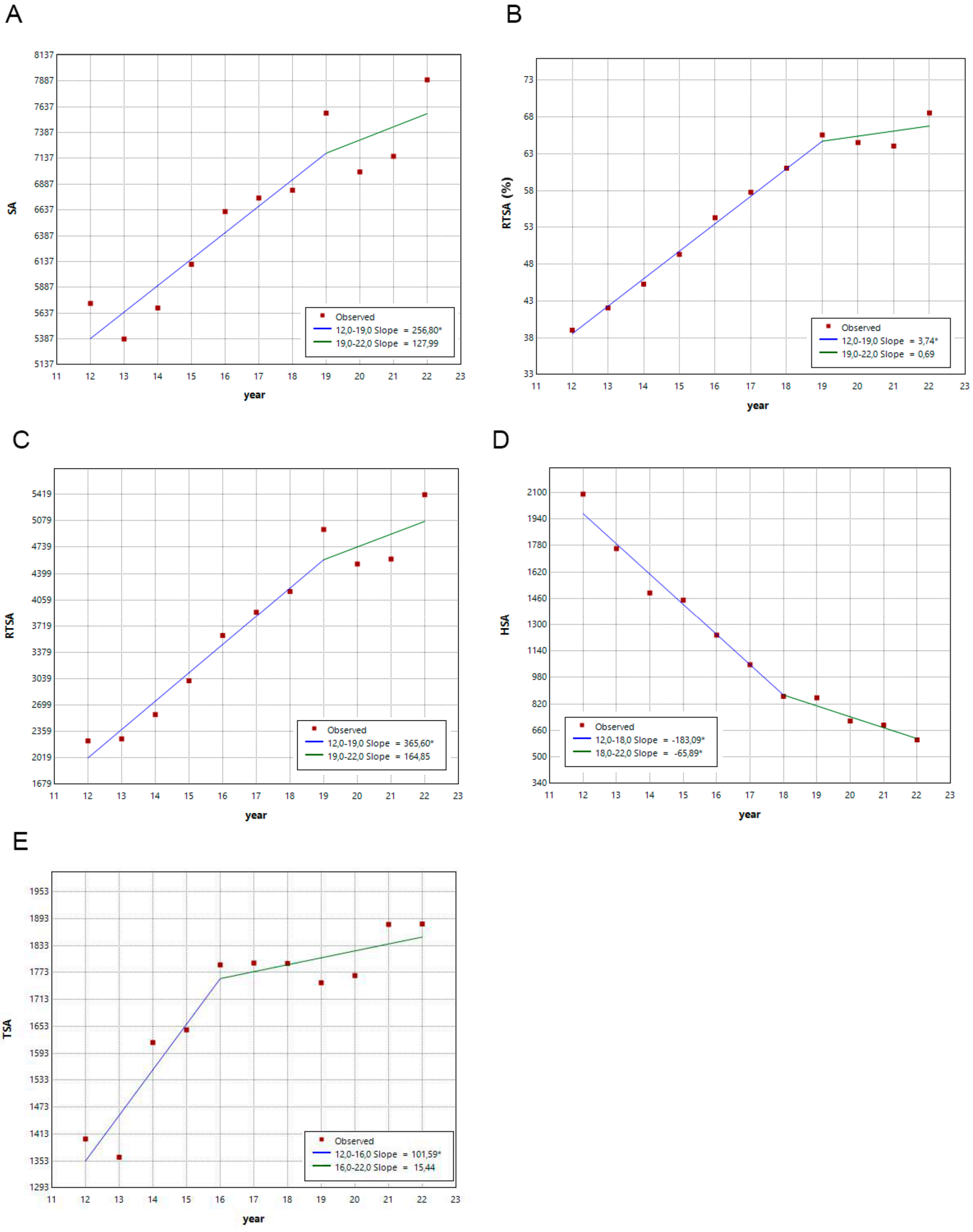


**Figure S1: Joint Point Regression Analysis.**

Joint Point Regression Analysis for All Shoulder Arthroplasties (SA; A), Ratio of Reverse Total Shoulder Arthroplasties (RTSA) to All Shoulder Arthroplasties (B), RTSA (C), Hemiarthroplasties (HSA; D), and Total Shoulder Arthroplasties (TSA; E) for the period 2012 to 2022. In the caption, inside the graph denotes the slope of the regression line. An asterisk (*) indicates a significant difference of the slope from zero ($p < 0.05$). The standard error of the slope is for figure A: 45.48 for 12-19 and 170.18 for 19-22, for figure B: 0.17 for 12-19 and 0.74 for 19-22, for figure C: 28.30 for 12-19 and 123.25 for 19-22, for figure D: 16.25 for 12-18 and 21.13 for 18-22, for figure E: 27.91 for 12-16 and 16.40 for 16-22.
